# Cr-Detector: A simple chemosensing system for onsite Cr (VI) detection in water

**DOI:** 10.1371/journal.pone.0295687

**Published:** 2024-01-03

**Authors:** Jyotsna Dei, Shirsak Mondal, Ayan Biswas, Dhruba Jyoti Sarkar, Soumyadeb Bhattacharyya, Souvik Pal, Subhankar Mukherjee, Subrata Sarkar, Alokesh Ghosh, Vipul Bansal, Rajib Bandhyopadhyay, Basanta Kumar Das, Bijay Kumar Behera

**Affiliations:** 1 Aquatic Environmental Biotechnology and Nanotechnology Division, ICAR-Central Inland, Fisheries Research Institute, Kolkata, West Bengal, India; 2 Department of Instrumentation and Electronics Engineering, Jadavpur University Salt Lake Campus, Kolkata, India; 3 Agri and Environmental Electronics (AEE) Group, Centre for Development of Advanced Computing (C-DAC), Kolkata, West Bengal, India; 4 Ian Potter NanoBioSensing Facility, NanoBiotechnology Research Laboratory, School of Science, RMIT University, Melbourne, Victoria, Australia; 5 College of Fisheries, Rani Lakshmi Bai Central Agricultural University, Jhansi, Uttar Pradesh, India; Minia University, EGYPT

## Abstract

Due to the increase in urbanization and industrialization, the load of toxicants in the environment is alarming. The most common toxicants, including heavy metals and metalloids such as hexavalent Chromium, have severe pathophysiological impacts on humans and other aquatic biotas. Therefore, developing a portable rapid detection device for such toxicants in the aquatic environment is necessary. This work portrays the development of a field-portable image analysis device coupled with 3,3’,5,5’-tetramethylbenzidine (TMB) as a sensing probe for chromium (VI) detection in the aquatic ecosystem. Sensor parameters, such as reagent concentration, reaction time, etc., were optimized for the sensor development and validation using a commercial UV-Vis spectrophotometer. The chemoreceptor integrated with a uniform illumination imaging system (UIIS) revealed the system’s applicability toward Cr(VI) detection. The calibration curve using the R-value of image parameters allows Cr(VI) detection in the linear range of 25 to 600 ppb, which covers the prescribed permissible limit by various regulatory authorities. Furthermore, the adjusted R^2^ = 0.992 of the linear fit and correlation coefficients of 0.99018 against the spectrophotometric method signifies the suitability of the developed system. This TMB-coupled field-portable sensing system is the first-ever reported image analysis-based technology for detecting a wide range of Cr(VI) in aquatic ecosystems to our knowledge.

## Introduction

Industrial advancement has played a vital role in improving human life while presenting environmental challenges. One such challenge faced by our societies and the sustainability of our planet is the ongoing release of toxic heavy metals and metalloids into the aquatic environment. The toxic metalloids have been infiltrating our environments through industrial discharge, mining activities, and domestic discharge into surrounding water bodies, thus polluting the aquatic ecosystem [1]. Through bioaccumulation and bio-amplification across food chains, these heavy metal species have significantly influenced biodiversity and life on Earth [2]. Chromium (Cr), present in the effluents from several industrial activities, such as chemical synthesis, chrome plating, leather tanning, production of dyes, metallurgy, mining, and manufacturing of various alloys, has become one of the most toxic heavy metals in our environments. Because of their high toxicity, chromium and its compounds, commonly utilized in industrial chemical processes, have become environmental contaminants of genuine concern [3]. A massive influx of chromium pollution in soil, groundwater, and air due to industrial activities has been associated with an elevated risk of carcinogenicity, mutagenicity, chromosomal breakage, DNA damage, and genotoxicity, which are responsible for a range of debilitating diseases [4]. The Cr(VI) contamination was observed in higher concentrations in Changhua County compared to Taichung City, resulting in 60 times more gastric cancer incidence in the former compared to Taichung City. Overexposure to Cr(VI) was observed in China, where upper stretches of rivers such as the Luo and Sandao exceeded Cr(VI) permissible levels, whereas lower and middle stretches did not [5]. Similar reports of chromium contamination of water supplies have been registered globally from USA [6], Australia, India [7], Bangladesh [8], Greece [9], Japan [10], and Iraq [11], and many resulting in successful lawsuits.

Chromium has two stable oxidation states viz. Cr(III) and Cr(VI), of which Cr(VI) is more soluble, mobile, and bioavailable in alkaline to slightly acidic conditions and, as a result, has a higher level of toxicity [12]. Cr(VI) is also regularly found in drinking and public water systems. In 1991, as a part of the Safe Water Drinking Act (SFDA), the United States Environmental Protection Agency placed chromium under the maximum contaminant level goal to have a maximum contaminant level of 100 ppb total chromium. However, even though it is recognized that Cr(VI) is significantly toxic while Cr(III) is not, the US EPA has not yet been able to establish MCL specifically for Cr(VI). However, according to research from the US National Toxicology Program, California could establish a state-wide drinking water standard of 10 ppb MCL for Cr(VI), under further review [13]. In addition, Japan and China also regulate Cr(VI). The chromium is detected by traditional laboratory tests using AAS [14] and ICP-MS [15]. For instance, for regulatory compliance, the total chromium in water is currently measured by ICP-MS using the US EPA Method 200.8 [16]. These tests are sensitive and reliable but require expensive equipment with a large footprint and trained technicians, making on-site detection challenging and delaying decision-making. Importantly, these assays do not allow specific detection of toxic Cr(VI). A rather complex anion-exchange chromatography-based US EPA Method 218.7 can be followed [6]. These limitations have led to an emerging interest in developing new methods for specific Cr(VI) detection in a point-of-care (POC). Notably, very few reported methods demonstrated the ability to detect Cr(VI) selectively. These methods have utilized various transducer/detection platforms, including fluorescence spectrometry [17] electrochemistry [18], and others. Among different transducers, colorimetric detection offers the key advantage of more practical deployment due to the ease of analyte detection by an unaided eye, as is evident from the success of COVID rapid antigen test (RAT) kits, and other standard POC devices such as pregnancy strips. Thus, colorimetric methods offer the opportunity to develop low-cost portable devices for rapid onsite monitoring of important target analytes, both qualitatively and semi-quantitatively. Chromium monitoring using the colorimetric method based on 1, 5-diphenylcarbazide dye is a well-known process [19], but over time it has faced many interference issues, and the better chromogenic substrate is well sought.

**Fig 1:**
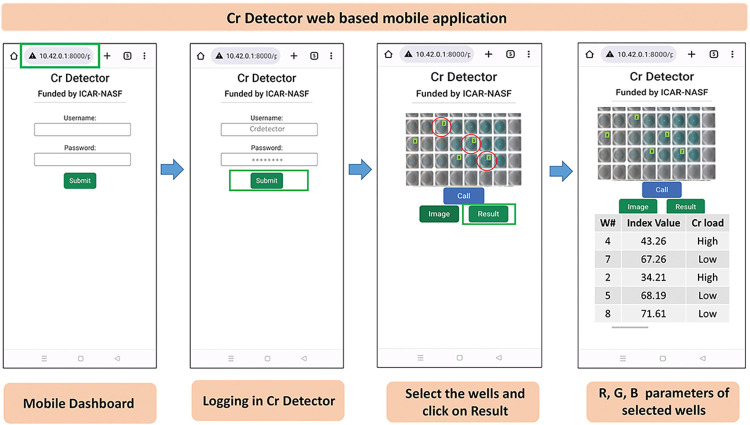
Graphical User Interface (GUI) and software operation flow diagram.

The conventional analysis of Cr(VI) in water requires highly skilled staff, time-consuming sample pre-treatment, and sophisticated equipment, including AAS, ICP-MS, UV-Vis spectrometry, surface-enhanced Raman scattering spectroscopy, fluorescence, electrochemistry, and X-ray Fluorescence, etc. which are more specific and sensitive towards minute concentrations of Cr(VI) [20,21]. But, detection through conventional systems is costly, requires skilled human resources for device and software handling, is time-consuming, and lacks field portability. These drawbacks of the conventional system have encouraged the development of inexpensive sensor-based rapid detection systems with high field portability and sensitivity. The precise detection of toxic metalloids using suitable sensing techniques may also flourish their separation and elimination techniques towards increasing the safety status of the usable water [22]. Moreover, the sensor development study may also be integrated with novel nanomaterials and quantum dots [23] to increase the stability and sensitivity of the developed sensor. Not only that, but such sensors can also be used in conventional adsorption studies towards developing novel adsorption beds in water treatment plants, as mentioned by Ismail et al. [24]. In the present study, a portable field device has been developed by exploiting TMB as a chromogenic substance. The first part of this endeavor depicts the choice of material through UV-Vis Spectroscopy. Furthermore, after establishing chemosensor selectivity and specificity towards Cr (VI), UIIS^Scan^1.1, an advanced image array technology-based field-portable high-throughput sensory system, was [25] exploited towards a simple, rapid and on-site analysis of Cr(VI). The tailored system has been upgraded and trained with an image-processing algorithm. A software named ‘Cr-Detector’ was developed for this purpose (**Fig 1**).

## Materials and methods

This section outlines experimental design details performed experiments and data analysis techniques. At first, suitable chromogenic compounds are selected based on the observable and measurable color change with different concentrations of Cr(VI). Next, different image analysis algorithms have been explored, and suitable color analysis techniques have been finalized for data collection. Furthermore, experimentations of different concentrations of Cr(VI) with suitable chromogen have been performed, and optimized reaction parameters. Next, repetitive experimentation with all optimized reaction parameters and selected concentrations of Cr(VI) have been executed towards cumulative data collection and standard curve generation. Different image parameters have been extracted from the images captured through the portable field device and evaluated towards selective output signal generation and finalization of the algorithm towards accurate sample analysis and validation. Further selectivity study of the developed sensor with different positive and negative ions has also been performed. Furthermore, recovery analysis and accurate sample analysis have also been executed.

### Reagents

3,3′,5,5′-tetramethylbenzidine (TMB), o-Phenyldiamine (OPD), 2,2’-azino-bis (3-ethylbenzothiazoline-6-sulfonic acid) (ABTS) hydrogen peroxide (H_2_O_2_, 30%), and DMSO, were obtained from Sigma-Aldrich. In addition, potassium dichromate (K_2_Cr_2_O_7_), Chromium chloride hexahydrate (CrCl_3_.6H_2_O), Zinc sulphate (ZnSO_4_), Lead acetate trihydrate (Pb(C_2_H_3_O_2_)_2_.3(H_2_O)), Sodium sulfate (Na_2_SO_4_), Potassium carbonate (K_2_CO_3_), Sodium hydrogen carbonate (NaHCO_3_), Barium nitrate (Ba(NO_3_)_2_), Cadmium nitrate tetrahydrate (Cd (NO_3_)_2_ · 4H_2_O), Mercury chloride (HgCl_2_) were purchased from HiMedia. Na_2_SO_4_ and ZnSO_4_ were used to obtain SO_4_^2-^ ions which were further used to check the specificity/cross-reactivity of the developed sensor intended to detect Cr(VI). In this article, water and other aqueous solutions with ultrapure water were purified with a Millipore system (18.2 MΩ·cm).

### Instruments and apparatus

Previously, an uniform illumination imaging system (UIIS) integrated with a portable scanner was reported that, allows the detection of an analyte through Mahanalobis Distance calculations [25]. The UIIS system can capture images of 96 well plates in 600 dpi resolution and operate at a wide temperature range, making it effective for spot experimentations in fields. The present study used a similar UIIS system with further modification. For capturing an image of the reaction colour, a different image analysis technique was explored to develop the algorithm for chromium detection. Here we have collected the ‘R’, ‘G’, and ‘B’ values of the reaction colour that signifies the ‘RED’, ‘GREEN’, and ‘BLUE’ parameters. Then we tried to find a predictive pattern with different colour parameters toward developing the standard curve. 96-well plates used in the study were obtained from Costar®, Corning Incorporated. For comparison with commercial spectrophotometer absorbance at a specific wavelength was measured by CLARIOstar^*plus*^, BMG Labtech.

### Reaction parameter optimisation

Different parameters regarding the Cr(VI) mediated oxidation of TMB were optimised initially. The optimisation of TMB concentration and H_2_O_2_ concentration was done by single variable method and using various concentrations of the reagents. The reaction time optimisation was performed in the Cr-Detector system.

### TMB concentration optimization

The TMB concentrations ranging from 0.1 to 1.5 mM were used for the above experiment. A fixed concentration of H_2_O_2_ and Cr(VI) was then added to the reaction mixture. Finally, the volume was adjusted to 200 μL with ultrapure deionised water. The absorbance maxima values at 650 nm of the concentrations were recorded.

### H_2_O_2_ concentration optimisation

The different concentrations of H_2_O_2_ (10 to 300 mM) were used for the optimisation experiment. At first, a fixed concentration of TMB was added to the wells, followed by different concentrations of H_2_O_2_ and a fixed concentration of Cr(VI). Next, the reaction volume was adjusted to 200 μL with ultrapure deionised water. Finally, the absorbance maxima values at 650 nm were recorded.

### Reaction time optimization

The reaction time was optimised with fixed TMB, H_2_O_2_ concentration, and different Cr(VI) concentration ranges from 0 to 1000 ppb. In addition, a kinetics study of the reaction was performed for 25 minutes with a time interval of 5 minutes. The image parameter values were recorded in the Cr-Detector system every 5 minutes.

### Colorimetric determination of Cr(VI)

In the present study, different parameters have been optimized to get a proper signal for detecting Cr(VI) up to the permissible limit by EPA. Solutions of Cr(VI) with concentrations ranging from 1ppb to 1000ppb were formed by serial dilution to obtain the detectable linear range of the Cr(VI). Concentrations of 1 ppb, 25 ppb, 50 ppb, 75 ppb, 100 ppb, 200 ppb, 400 ppb, 600 ppb, 800 ppb and 1000 ppb were used for the test. The different concentrations of Cr(VI) were added to the reaction mixture containing optimum concentrations of TMB and H_2_O_2_. The reaction volume was then adjusted to 200 μL with ultrapure deionised water, and absorbance was recorded at 650 nm after the optimized incubation. Further, the mentioned colorimetric experiment for Cr(VI) detection was repeated 500 times in the developed Cr-Detector towards the development of the calibration curve.

### Specificity test

Solution of other metals and ions like Hg^2+^, Pb^2+^, Ba^2+^, Cr^3+^, As^3+^, Cd, SO_4_^2−^ CO_3_^2−^, and HCO^3−^ were prepared from their respective salts. A fixed concentration of these ions, along with Cr(VI), was added to optimum concentrations of TMB and H_2_O_2_. The reaction volume was adjusted to 200 μL with ultrapure deionised water followed by measuring absorbance at 650 nm. Further anti-interference tests were performed using a mixture of different metal ions in the same reaction mixture. All the mentioned experiments were performed in triplicates and repeated more than once.

## Results and discussion

### Choice of materials

**Fig 2:**
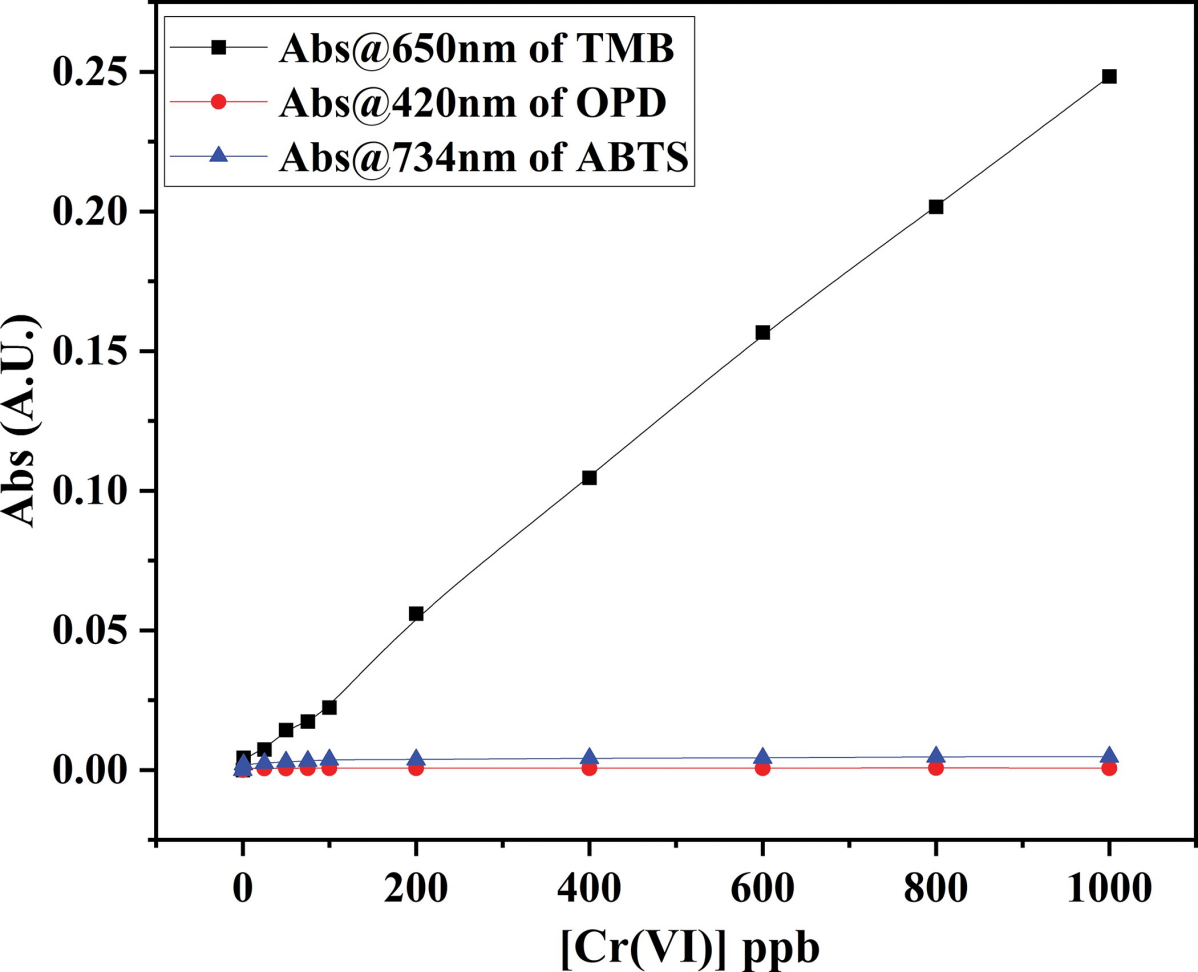
Choice of chromogen such as TMB, OPD and ABTS against Cr(VI) concentrations under the condition of H_2_O_2_ = 50 mM in UV-Vis Spectrophotometer.

Cr(VI) and Cr(III) redox conversion is ubiquitous in the environment. In other words, Cr(III) can easily be oxidised to Cr(VI) by an oxidant and Cr(VI) can quickly be reduced to Cr(III) by a reductant [26]. Several attempts have been envisaged toward colorimetric Cr(VI) detection. One such testimony is 1,5-diphenylcarbazide (DPC), which can produce a strong colour change for quantitative or semi-quantitative analysis [27,28]. The DPC-based sensor is one the favourites among several other chromogens for environment monitoring. Despite its advantages, the use of DPC is limited due to its unstable nature, reduced sensitivity, and narrow linear range [29]. Research works have been conducted to increase the stability of DPC by coating various polymers, which, moreover, increases the sensor fabrication cost. Therefore, in this study, other different chromogenic substances such as 3,3’,5,5’-tetramethylbenzidine (TMB), o-Phenyldiamine (OPD), 2,2’-azino-bis (3-ethylbenzothiazoline-6-sulfonic acid) (ABTS) are envisaged towards cost effective colorimetric detection of Cr(VI). 3,3′,5,5′- tetramethylbenzidine (TMB) is one of the most used chromogenic substrates among peroxidase probes. In the presence of a catalyst, TMB can be oxidised by H_2_O_2_ to oxidised TMB forming intense blue colour, which can be visualised through the naked eye and spectroscopy. The absorbance peak can be recorded at 650 nm [30]. On the other hand, o-phenylenediamine (OPD) is a chromogenic substrate that generates yellow colour upon oxidation. The oxidation by H_2_O_2_ results in the formation of 2, 3-phenazinediamin, generating bright yellow colour and yellow fluorescence. The absorbance maxima of the colour change were measured at 420 nm [31]. Similarly, ABTS is a chemically stable and highly water-soluble peroxidase substrate. When oxidised by H_2_O_2_, it serves as a peroxidase substrate and creates a metastable cation by forming ABTS^4+^. The oxidation produces blue-green colour, which can be quantified by recording absorbance maxima at 734 nm [32]. This work chose TMB as the chromogenic substrate because of its superior sensitivity over OPD and ABTS comparatively. For example, a fixed concentration of the OPD and ABTS failed to produce a measurable colour change for different Cr(VI) concentrations in the presence of 50 mM H_2_O_2_, while the same concentration of TMB produced excellent measurable colour changes in the presence of 50mM H_2_O_2_ (**Fig 2**). Also, TMB was found as the chosen substrate in almost every literature regarding peroxidise, nanozyme and DNAzyme activities [33].

### Reaction parameter optimisation

#### TMB concentration optimization

The absorbance maxima values at 650 nm of the TMB concentrations of 0.1 mM to 1.5 mM were found to be in a range of 0.0047 to 0.0685. It was observed that, from 0.9 mM concentration, the absorbance values got saturated. Therefore, as per this study, 0.9 mM concentration of TMB was found to be optimum for further experiments. The graph for TMB concentration optimisation is represented in **S1 Fig in S1 File.**

### H_2_O_2_ concentration optimisation

The absorbance maxima values at 650 nm of the H_2_O_2_ concentrations of 10 mM to 300 mM were found in a range of 0.0243 to 0.1523. From the absorbance maxima values, it was observed that, the absorbance values get saturated from H_2_O_2_ concentration of 50 mM to 300 mM. Thus, 50 mM H_2_O_2_ was finalized as the optimum H_2_O_2_ concentration for further experimentations. The graph for H_2_O_2_ concentration optimisation is represented in S2 Fig in S1 File.

### Colorimetric detection of Cr(VI) through spectrophotometer

**Fig 3:**
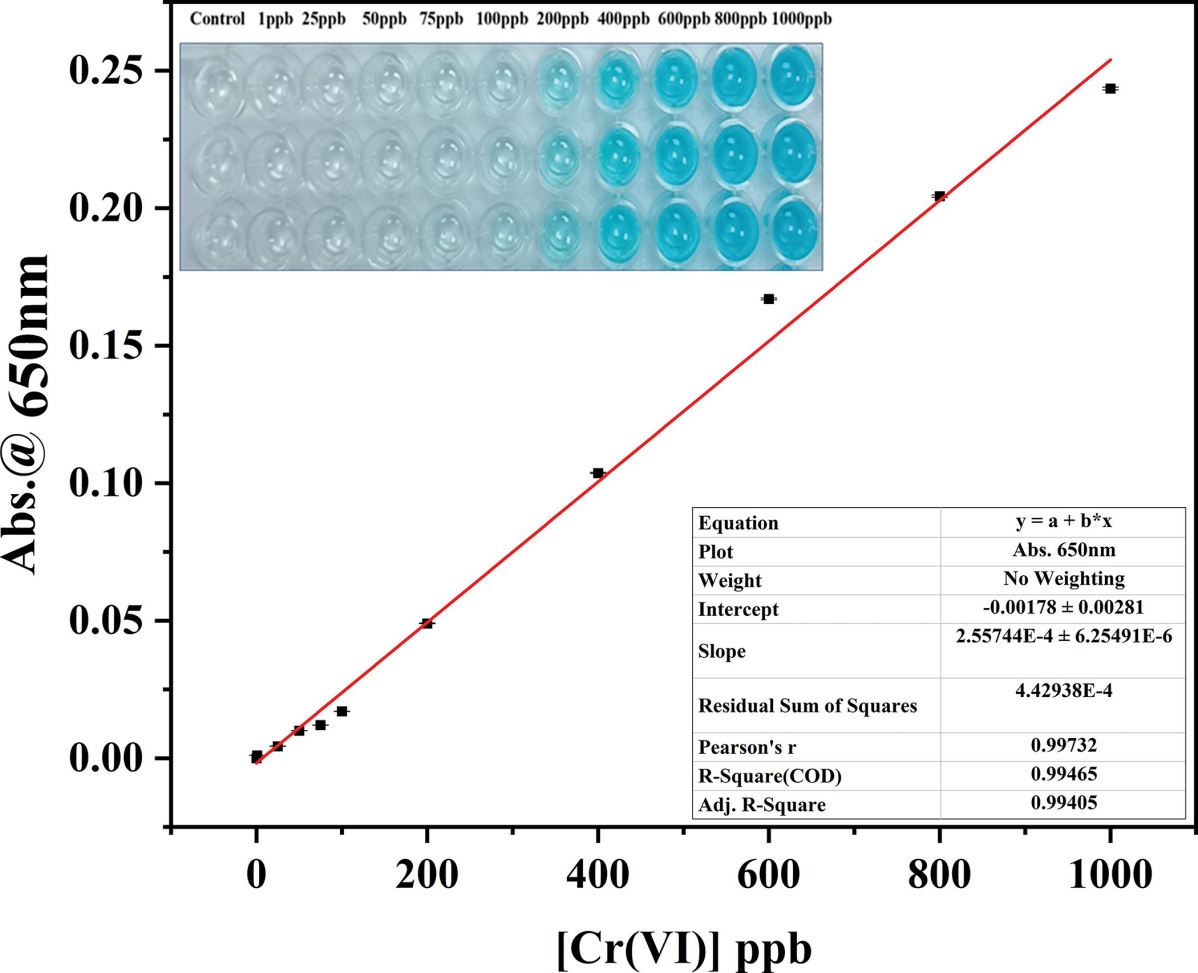
Calibration curve of Cr(VI) under the optimized condition of [TMB] = 900 μM and [H_2_O_2_] = 50mM using UV-Vis Spectrophotometer; Captured image of visual colour change against different [Cr(VI)] (Inset).

In this work, the enzyme-linked immunosorbent assay (ELISA) substrate 3,3′,5,5′-Tetramethylbenzidine (TMB) is utilized as a probe for the colorimetric detection of Cr(VI). The sensing mechanism of this colorimetry is based on the oxidation of TMB by Cr(VI) in an acid media with the assistance of H^+^ ions produced by H_2_O_2_. TMB is converted to oxidized TMB when Cr(VI) is reduced to Cr(III) in an acidic environment. When TMB is oxidized from its colorless form, it becomes blue. Maximum absorption for this blue color occurs at 650 nm wavelength. As a result, by measuring absorbance at 650 nm, the intensity of the generated color is assessed spectroscopically. The absorbance maxima values at 650 nm of different Cr(VI) concentrations of 1 ppb to 1000 ppb were found to be in the range of 0 to 0.2435. Furthermore, it was found that, as the concentration of Cr(VI) increased, the absorbance at 650 nm increased steadily. The inset of **Fig 3** provides images of the TMB-H_2_O_2_ solutions mediated by various Cr(VI) concentrations for naked-eye examination. The linear fit analysis for the standard curve depicted excellent goodness of fit value of 0.994 (**Fig 3**). The equation for the standard curve is as follows:

$$Y_{1}=m_{1}\times X_{1}+c_{1}$$

Where ‘Y_1_’ is the absorbance value at 650 nm, ‘m_1_’ is the slope, i.e., 2.55744 x 10^−4^ ± 6.25491 x 10^−6^, ‘X_1_’ is the Cr(VI) concentration and ‘c_1_’ is the intercept value, i.e., -0.00178 ± 0.00281.

### Effects of oxidized ions on specificity and anti-interference

To confirm the selective/specific response for detecting Cr(VI), numerous oxidized ions, including cations and anions (Cr^3+^, Pb^2+^, As^3+^, Hg^2+^, Cd^2+^, Ba^2+^, SO_4_^2-^, HCO^3-^, PO_4_^3-^) were supplemented in the reaction medium in presence and absence of Cr^6+^. The absorbance maxima values at 650 nm were recorded for several other ions in the same TMB-H_2_O_2_ reaction system. **S3 Fig in S1 File.** shows naked-eye color formation only in the wells where Cr(VI) was added. Also, there is a significantly high signal output in the case of Cr(VI), unlike other ions when the absorbance values were studied.

To further investigate the viability of the new colorimetric chemosensor for Cr(VI), an anti-interference experiment was conducted. Under the optimized experimental conditions, equal amounts of other metals and other ions were added to the reaction system, and the absorbance maxima values were then measured at 650 nm. As shown in **S3 Fig in S1 File**, the presence of interfering ions has no discernible effect on the absorbance intensity at 650 nm of TMB solution after adding Cr(VI). These results demonstrated the novel visual chemosensor’s excellent specificity and anti-interference for a qualitative Cr(VI) study.

### Development of UIIS sensing system for Cr(VI) detection

#### Image parameter selection

In this work, different image parameter values, such as ‘RED’ ®, ‘GREEN’ (G), and ‘BLUE’ (B), are extracted from the Cr-Detector system for further data analysis. When the G and B values are plotted against the Cr(VI) concentrations, the different Cr(VI) concentrations cannot be clearly distinguished based on either G or B values, as both the G and B values for different concentrations appeared to be similar and thus no distinguishable pattern is observed. The linear fit equations for the plot of Cr(VI) concentration vs. G value and Cr(VI) concentration vs. B value provide poor goodness of fit values of 0.50 and 0.26. In contrast, the R-value and R+G+B value expressed a distinguishable pattern. The R-value for each concentration of Cr(VI) appeared to be much more distinguished; the linear regression analysis provided an excellent adjusted-R^2^ value of 0.94. A similar pattern is observed for the R+G+B value vs. Cr(VI) concentration plot, where the linear fit with adjusted-R^2^ value is 0.84. From this, R-value was targeted among other image parameters for all the experiments of colorimetric detection of Cr(VI) due to its highest linearity and adjusted-R^2^ value. The image parameter selection is represented in **S4 Fig in S1 File.**

#### Reaction time optimization

The reaction time was optimised with fixed TMB, H_2_O_2_ concentration, and different Cr(VI) concentration ranges from 0 to 1000 ppb. Then, a kinetics study of the reaction is performed for 25 minutes with a minimum interval of 5 minutes. We considered the ‘RED’ ® value among the other image parameters for further analysis. The data generated every 5 minutes are plotted against the Cr(VI) concentration ranges from 0 ppb to 1000 ppb. Visual colour changes for the concentrations appeared from 15 minutes of the kinetics study. Before 15 minutes, the visual colour changes were negligible. The plot explained that the difference between the ‘R’ values of all the concentrations before 15 minutes of the kinetic study was significantly less and thus produced flat patterns with minimum slope. Whereas distinguishable differences appeared for the different concentrations from 15 to 25 minutes of the kinetic study, thus producing patterns with a higher slope. The linear regression analysis of the patterns observed at 15 minutes, 20 minutes and 25 minutes of the experiments showed adjusted goodness of fit values (R^2^) as 0.93, 0.90 and 0.86, respectively. From this, it was concluded that the reading could be taken in the Cr-Detector after 15 minutes of incubation of the reaction mixture to get the best possible output. The reaction time optimization through Cr-Detector is represented in **S5 Fig in S1 File.**

#### Colorimetric detection of Cr(VI) through Cr-Detector

**Fig 4:**
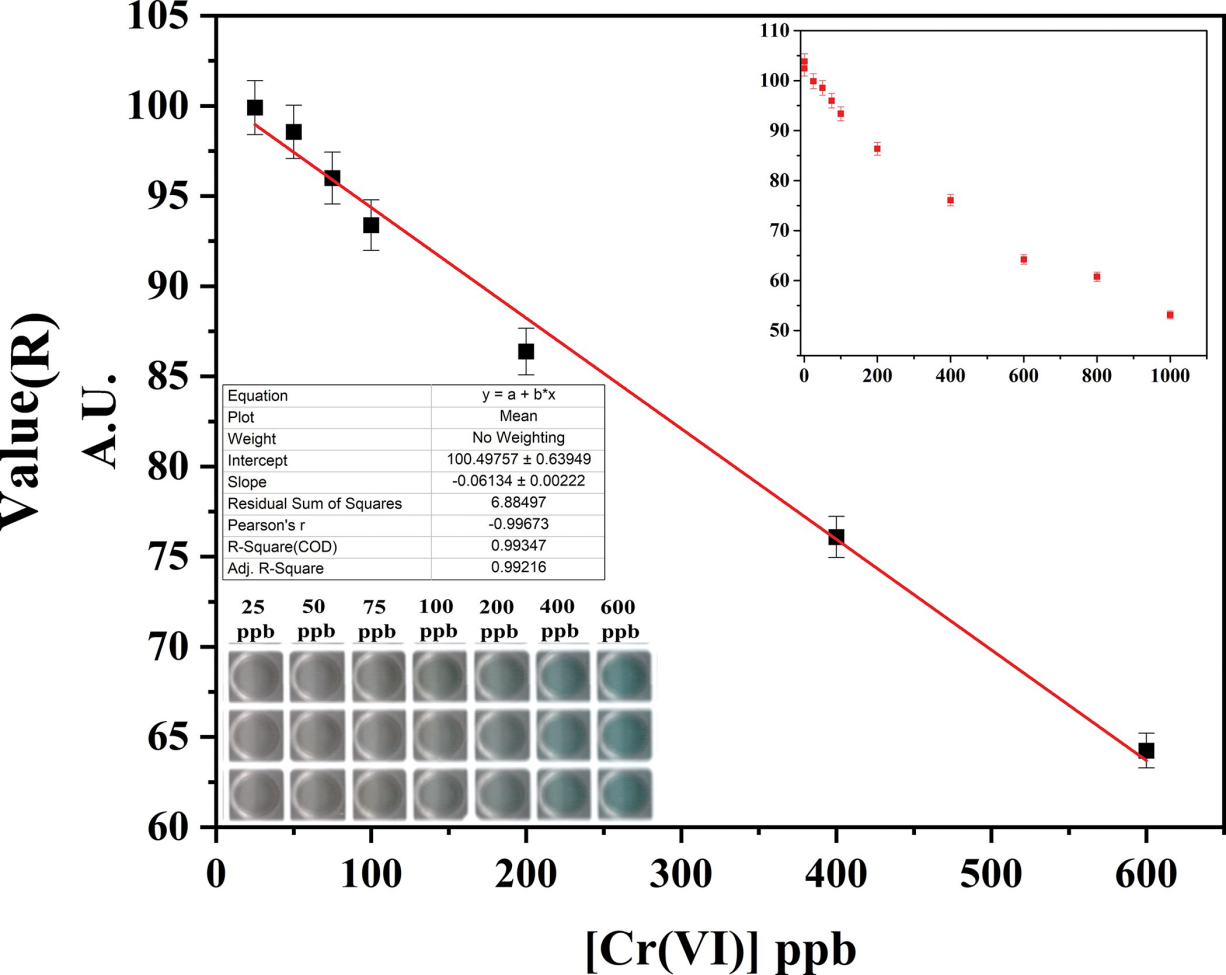
Calibration curve of various [Cr(VI)] under optimized parameters in Cr-Detector; Full experimental range of different [Cr(VI)] from 0 to 1000 ppb under optimized parameters (Inset).

Further, the mentioned protocol for colorimetric detection of Cr(VI) was repeated 500 times in Cr-Detector to generate the R-values of the image acquired through scanning the 96 well plates towards developing the calibration curve (**Fig 4**).

The average R-values of the concentrations range from 0 ppb to 1000 ppb and are plotted against the concentrations towards the development of the calibration curve (Represented in the inset of **Fig 4**). The plot demonstrates the calibration curve with the linearity found from 25 ppb to 600 ppb of Cr(VI).

The equation of the calibration curve is found to be:

$$Y_{2}=m_{2}\times X_{2}+c_{2}$$

Where ‘Y_2_’ is R-value generated in Cr-Detector, ‘m_2_’ is the slope, i.e., -0.06134 ± 0.00222, ‘X_2_’ is the Cr(VI) concentration, and ‘c_2_’ is the intercept value, i.e., 100.49757 ± 0.63949. The adjusted-R^2^ value is found to be 0.99216. The calculated theoretical Limit of Detection (LOD) and Limit of Quantification (LOQ) values from the calibration curve is 0.12 ppb and 0.36 ppb, respectively, with a linear detection range 25 to 600 ppb.

#### Recovery analysis

A recovery analysis has been carried out to check the device’s applicability. 50, 100, 250 and 500 ppb of Cr(VI) were spiked in Milli-Q water, and the recovery percentages of the spiked concentrations are 101.056%, 100.026%, 100.0324% and 99.9716%, respectively. The recovery error (RE) percentages for 50, 100, 250 and 500 ppb are -1.056, -0.026, -0.3024 and 0.0284, respectively (**S1 Table in S1 File**).

#### Real sample analysis

Further, 27 samples from the southern belt of river Hooghly of West Bengal, India, were collected where most industries are located. The samples were analyzed per the above reaction protocol, and the data were taken with the spectrophotometer and Cr-Detector. The data are represented in **Table 1**. Out of 27 samples, except 3, the rest of the samples’ detection was comparable with the conventional Spectrophotometric analysis. The correlation coefficient of the UIIS data with Spectrophotometer data was 0.99018.

**Table 1:** Comparison of predictive data after analysis of the river water sample through commercial UV-Vis Spectrophotometer and Cr-Detector.

Sample ID	Spectrophotometer		Cr-Detector		
		Predictive [Cr(VI)] (ppb)	UIIS Index (A.U.)	Predictive [Cr(VI)] (ppb)	Error percentage (%)
1	0.0005	20.643	86.213	232.875	10.281
2	0.0430	187.412	89.779	174.740	0.068
3	0.0716	299.569	83.092	283.752	0.053
4	0.1420	575.647	65.795	565.741	0.017
5	0.0001	19.176	N.D.*	N.D.	N.A.^#^
6	0.0251	117.255	93.702	110.785	0.055
7	0.1101	450.549	73.373	442.200	0.019
8	0.0448	194.471	88.976	187.831	0.034
9	0.0770	320.745	81.041	317.192	0.011
10	0.1160	473.686	71.234	477.072	0.007
11	0.0100	58.000	97.994	40.815	0.296
12	0.0260	120.745	93.523	113.703	0.058
13	0.0002	19.569	N.D.	N.D.	N.A.
14	0.0200	97.216	94.593	96.260	0.010
15	0.1130	461.922	72.660	453.824	0.018
16	0.0160	81.529	N.D.	N.D.	N.A.
17	0.0603	255.255	84.607	259.057	0.015
18	0.1010	414.863	75.959	400.042	0.036
19	0.0003	19.961	N.D.	N.D.	N.A.
20	0.0011	23.098	N.D.	N.D.	N.A.
21	0.1060	434.471	73.908	433.478	0.002
22	0.1400	567.804	66.686	551.216	0.029
23	0.1470	595.255	64.623	584.848	0.017
24	0.0308	139.569	75.200	412.416	1.955
25	0.0520	222.706	86.218	232.794	0.045
26	0.0280	128.588	93.345	116.605	0.093
27	0.0148	76.824	96.109	71.545	0.069

*N.D. = Non-Detectable (when, 25 ppb<predictive concentration>600 ppb)

^#^N.A. = Not Applicable.

### Comparison of ‘Cr-Detector’ with other sensors for detection of Cr(VI)

Lots of similar work on sensor development for the detection of Cr(VI) have been reported in earlier studies. The result obtained from ‘Cr-Detector’ was compared with other sensors and it correlates well with other reported literature in **Table 2**.

**Table 2:** Recent advancements in sensor-based detection of Cr(VI).

Developed sensor	Sensor design	Transduction principle	Linear Range	Limit of Detection	Ref.
Colorimetric paper-based analytical device	Diphenylcarbazide on μPAD	Optical	10–90 ppb	3 ppb	[34]
Wireless portable device	Using 1, 5 diphenyl carbazide as chromogen	Optical	-	2 ppb	[35]
POC device for on-the-spot detection of Cr(VI) in wastewater	Using citrate-capped silver nanoparticles	Optical	10–700 ppb	26 ppb	[36]
Photoelectrochemical sensor for detection of trace hexavalent chromium	By facilitating the photon-excited electron-hole pair separation coupled with light-sensitive quercetin	Photo-electrochemical	1.4–2592.3 ppb	0.44 ppb	[37]
Portable sensing device (Kavach) for non-invasive and selective detection of Cr(VI) in wastewater and living cells	Devicethin layer (~1 mm) of carbon dot decorated boehmite nanostructure incorporated in a layer of polyvinylidene fluoride-co-hexafluoropropylene membrane.	Optical	-	3.4 ppb	[38]
Cr(VI) detection with disposable screen-printed electrode modified with gold nanoparticles	Gold nanoparticle electrodeposition-based voltammetric detection.	electrochemical	10–5000 ppb	5 ppb	[39]
Gold-silver nanoparticles modified electrochemical sensor for determination of chromium (VI) in wastewater samples	Silver-gold bimetallic nanoparticles through electrochemical deposition	electrochemical	50–5000 ppb	0.1 ppb	[40]
Electrochemical detection of Cr(VI) in water sample by using modified glassy carbon electrode (GCE)	Graphene carbon nitride decorated with silver molybdate immobilized with nafion modified glassy carbon electrode.	electrochemical	5.199–36.39 ppb	0.0831 ppb	[41]
Flower-like self-assembly of gold nanoparticles (AuNPs) on a glassy carbon electrode (GCE) for detection of chromium (VI)	The first is sol–gel film derived from 3-mercaptopropyltrimethoxysilane (MPTS). The second AuNP layer is then self-assembled on the surface of the sol–gel film, forming flower-like gold nanoelectrodes enlarging the electrode surface.	electrochemical	0.01–1.2 ppb	0.0029 ppb	[42]
Electrochemical detection of Chromium(VI) using NiO nanoparticles	Sol–gel synthesized Nickel Oxide (NiO) nanoparticles coated onto the fuorine doped tin oxide plate	Optical	5000–50000 ppb	5000 ppb	[43]
Electrochemical detection of Cr(VI) based on gold nanoparticle decorated titania nanotube arrays	Au-nanoparticle-decorated titania nanotubes (TiO_2_NTs) grown on a titanium substrate	electrochemical	5.199–5448 ppb	1.55 ppb	[44]
Electrochemical detection of sub-ppb level chromium(VI) using nano-sized gold particle	Nano-sized Au particles grown on a conducting substrate modified with sol–gel-derived thiol functionalized silicate network	electrochemical	0.2–3 ppb	0.1 ppb	[45]
A miniaturized portable electrochemical system for Cr(VI)	Screen-printed carbon electrode with gold nanoparticles modification	electrochemical	20–200 ppb	5.4 ppb	[46]
Electrodeposited AuNPs/rGO Nanocomposite as sensor for Cr(VI) determination in Water	Electrodeposition of reduced graphene oxide (rGO) on GCE by electroreduction of GO using cyclic voltammetry (CV), and AuNPs were electrodeposited on rGO sheets using CV to obtain as AuNPs/rGO/GCE	electrochemical	5.19–1559 ppb	2.39 ppb	[47]
Ion-imprinted chitosan-graphene nanocomposites for the determination of Cr(VI)	Ion-imprinted polymers electrodeposition of chitosan-graphene nanocomposites (IIP-S)	electrochemical	0.051–520 ppb	0.33 ppb	[48]
‘Cr-Detector’ system for specific detection of Cr(VI) in water	Image parameter extraction and analysis for TMB oxidation by Cr(VI) in presence of H_2_O_2_	Optical	25–600 ppb	0.12 ppb	Present study

## Conclusion

Herein we report the development of a chemo-sensor, TMB, for detecting Cr(VI) in water using the spectrophotometric method. The sensor was tested against various interfering parameters. In the presence of those interfering substances, the chemo-sensor exhibited excellent specificity and selectivity towards Cr(VI). Using the spectrophotometric method, the linear range was 1 to 1000 ppb with an adjusted-R^2^ value of 0.994. Furthermore, the chemo-sensor was integrated with Cr-Detector to demonstrate a lab-to-field application that, extended the sensing system’s applicability. Using a uniform illumination-based system, the linear range was found to be 25 to 600 ppb of Cr(VI) with LOD 0.12 ppb, which is well comparable with other reported literature concerning the linear range and detection limit, as presented in **Table 1**.

Furthermore, the exhibited wider linear range covers the prescribed permissible limit by USEPA and the Food Safety and Standards Authority of India (FSSAI). Real sample analysis revealed a good correlation with spectrophotometric data. Integrating TMB as a chemo-sensor and Cr-Detector, a selective, rapid, portable, user-friendly device for measuring Cr(VI) is first reported in this endeavor, to our knowledge. Image analysis-based field portable sensor and sensing system development for low-cost rapid onsite monitoring of environmental toxicants will encourage the future development of array-based sensor integrated devices that will enable cumulative detection and multi-parametric analysis of more than one analyte at a time. Moreover, the advancement in soft computing has revolutionized data analytics for sensor development. The rapid emergence of deep learning has been successfully applied to improve the analytical performance of colorimetric determination (e.g., UV-Vis spectrophotometer and colorimetric test paper), including denoising, recognition, and summary of every small characteristic change from each image [49–52]. One example is Deep Convolutional Neural Networks (DCNNs) [51]. DCNNs have excellent potential to increase the sensitivity and selectivity of any biosensor by rapid and effective decision generation. In comparison to human methods, the automated aspect of DCNNs enables quick and effective analysis. The rapid onsite one-click picture processing can be achieved through DCNNs. Moreover, Users with different degrees of image analysis or colorimetric sensing experience can utilize this one-click method since it is user-friendly and accessible. Again, Few-Shot Learning (FSL) and Generative Adversarial networks (GAN) can increase the accuracy of DCNNs. These soft computing technologies may be explored to develop effective decision support systems with superior sensitivity and selectivity in future.

## Supporting information

S1 File Supporting information contains supporting figures and table. (DOCX)
