# Supplementary material for: Cr-Detector: A simple chemosensing system for onsite Cr (VI) detection in water
Source: PLoS One. 2024 Jan 3;19(1):e0295687. doi: 10.1371/journal.pone.0295687 (PMC10763940; doi:10.1371/journal.pone.0295687)
Supplement: S1 File — (DOCX) [file pone.0295687.s001.docx]

**Supporting Information**

**Cr-Detector: A simple chemosensing system for onsite Cr (VI) detection in water**

Jyotsna Dei^1,2^, Shirsak Mondal^1^, Ayan Biswas^1^, Dhruba Jyoti Sarkar^1^, Soumyadeb Bhattacharyya^3^, Souvik Pal^3^, Subhankar Mukherjee^3^, Subrata Sarkar^3^, Alokesh Ghosh^3^, Vipul Bansal^4^, Rajib Bandhyopadhyay^2^, Basanta Kumar Das^1^, Bijay Kumar Behera^1,5^*

^1^Aquatic Environmental Biotechnology and Nanotechnology Division, ICAR-Central Inland

Fisheries Research Institute, Kolkata-700120, West Bengal, India

^2^Department of Instrumentation and Electronics Engineering, Jadavpur University Salt Lake

Campus, Block LB, Sector III, Salt Lake, Kolkata-700098, India

^3^Agri and Environmental Electronics (AEE) Group, Centre for Development of Advanced

Computing (C-DAC), Sector-V, Salt Lake, Kolkata, West Bengal-700091, India

^4^Ian Potter NanoBioSensing Facility, NanoBiotechnology Research Laboratory, School of

Science, RMIT University, Melbourne, Victoria 3001, Australia

^5^College of Fisheries, Rani Lakshmi Bai Central Agricultural University, Gwalior Road, Near Pahuj Dam, Jhansi-284003, Uttar Pradesh, India

***Corresponding author**

Dr. Bijay Kumar Behera

Dean

College of Fisheries

Rani Lakshmi Bai Central Agricultural University,

Gwalior Road, Near Pahuj Dam, Jhansi-284003, Uttar Pradesh, India

Email: beherabk18@yahoo.co.in

Tel: 0510-2730040 (Ext: 7203)


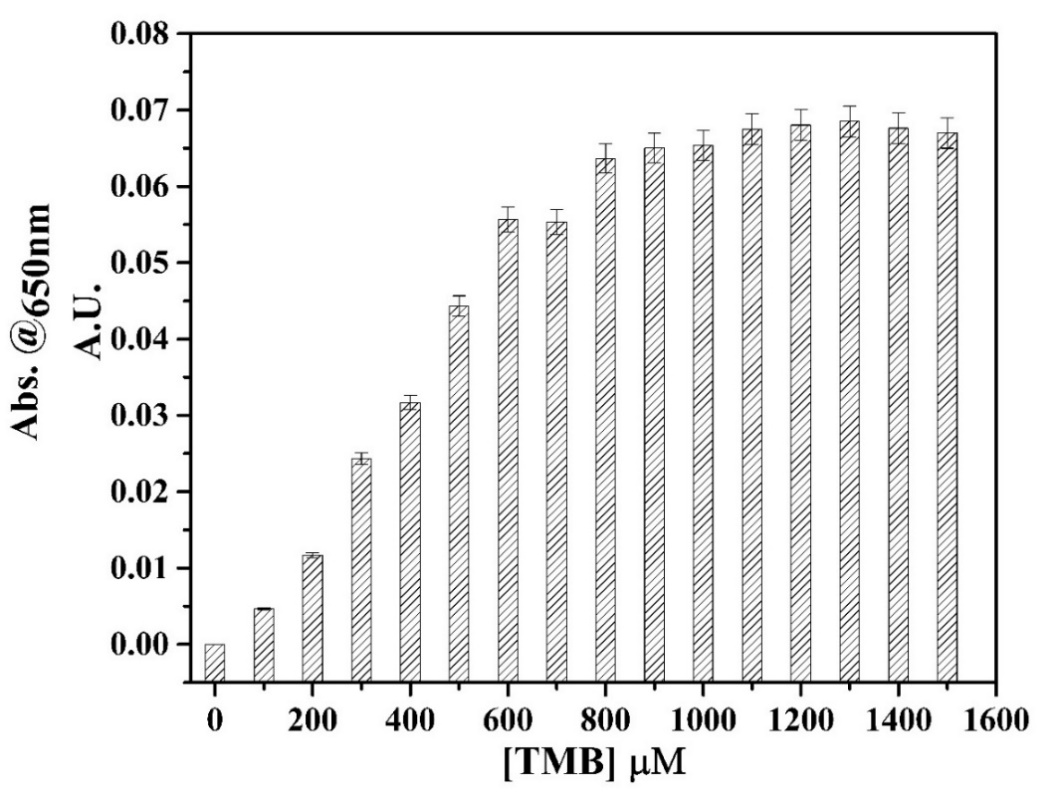


**S1 Fig.** TMB concentration optimization for Cr(VI) detection through UV-Vis spectrophotometer.


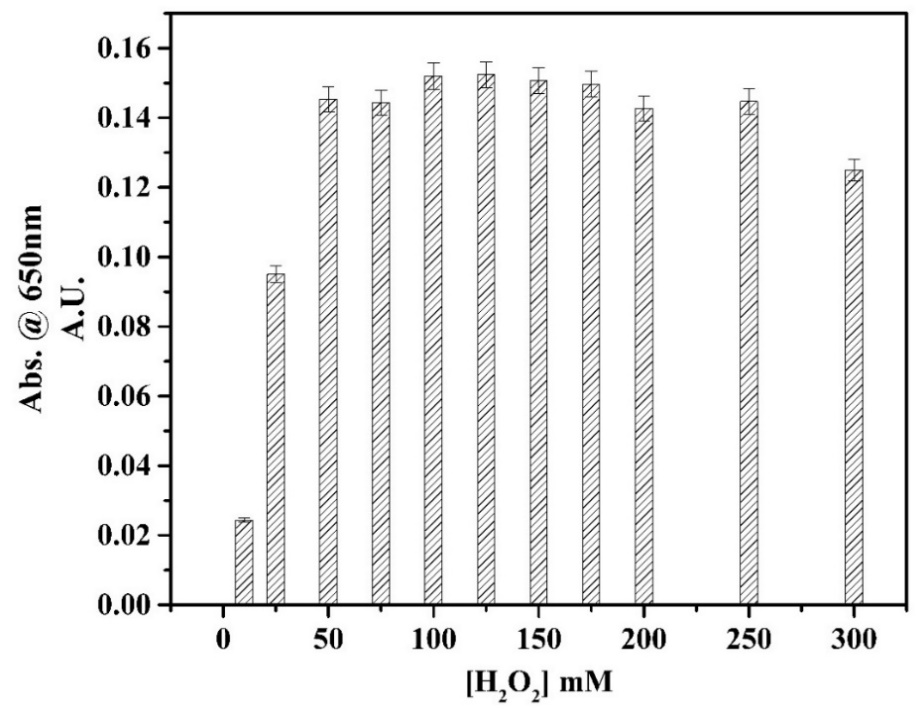


**S2 Fig.** H_2_O_2_ concentration optimization for Cr(VI) detection through UV-Vis spectrophotometer.

**

** **S3** **Fig.** Specificity analysis of Cr (VI) through TMB oxidation in UV-Vis spectrophotometer; (a) Specificity index vs. different analytes; (b) Specificity index vs. mixture of individual analytes with Cr (VI).

**S4 Fig**. Image parameter selection for Cr (VI) detection through Cr-Detector: (a) plot of Cr (VI) concentration vs. different image parameter values such as R, G, B, and R+G+B (inset of a); (b) Linear fitting curve of Cr (VI) concentration vs. R+G+B value plot and goodness of fit calculation; (c) Linear fitting curve of Cr (VI) concentration vs. R-value plot and goodness of fit calculation.

**S5 Fig**. Reaction time optimization for Cr (VI) detection through Cr-Detector: linear regression analysis of Cr (VI) concentration vs ‘R’ value plot at different incubation times (15 min, 20 min & 25 min). The inset represents scatter plot of Cr (VI) concentration vs ‘R’ value at different incubation times (T=0 min, 5 min, 10 min, 15 min, 20 min, 25 min).

**S1 Table.** Recovery analysis of Cr (VI) in Cr-Detector

| **[Cr (VI)] Added (ppb)** | **Developed device** | | |
| --- | --- | --- | --- |
|  | **[Cr (VI)] Found (ppb)** | **RE (%)** | **Recovery (%)** |
|  | **Mean ± SD** |  | |
| 50 | 50.528 ± 0.79 | -1.056 | 101.056 |
| 100 | 100.026 ± 0.99 | -0.026 | 100.026 |
| 250 | 250.756 ± 0.84 | -0.3024 | 100.3024 |
| 500 | 499.858 ± 1.69 | 0.0284 | 99.9716 |
